# Subretinal fluid application to close a refractory full thickness macular hole

**DOI:** 10.1186/s40942-017-0094-7

**Published:** 2017-11-27

**Authors:** Carsten H. Meyer, Robert Borny, Nicole Horchi

**Affiliations:** Pallas Clinics, Bahnhofplatz 2, 5000 Aarau, Switzerland

## Abstract

**Background:**

To close a refractory full thickness macular hole (FTMH) by adjacent subretinal fluid application to release the elastic retina from the retinal pigment epithelium (RPE).

**Case presentation:**

A 83 years old patient presented an old FTMH with a diameter of 1444 μm. After confirming intraoperatively the complete release of the epiretinal membrane around the FTMH, we installed 3 small subretinal blebs around the hole, to release the adjacent retina from the RPE. The mobilized retina was gently moved towards the macular center. A silicone oil tamponade was installed to secure a proper healing and observation of the FTMH. The closure of the 1444 μm FTMH was seen on indirect ophthalmoscopy and confirmed by OCT 5 days after surgery by restoring the retinal architecture. A late reopening was not apparent at the postoperative observations. Visual acuity improved from hand motion to 20/200 at 4 weeks postoperative.

**Conclusion:**

Although FTMH develop by epiretinal tangential traction, large FTMH may persist even after complete release of its epiretinal traction. Subretinal fluid application may release the flexible retina from the RPE to achieve a relocation at the central fovea facilitating an anatomical closure of the macular hole.

## Background

Full thickness macular hole (FTMH) is a defect of all neuroretinal layers at the foveal center. Treatment of FTMH by pars plana vitrectomy (PPV) and consecutive gas tamponade was first described by Kelly and Wendel and has been refined since then, to improve the anatomical functional outcome [1]. The current standard approach treats the underlying epiretinal pathology by removing vitreous adhesions including the internal limiting membrane (ILM) to release tangential traction from the epiretinal surface. An additional intraocular gas tamponade will assist the healing and closure of the macular hole within days. With this approach, most authors report anatomical closure rates greater than 90%, although some MHs fail to close following this approach [2].

Attempts to treat refractory macular holes may include installation of silicone oil, application of autologous platelet concentrates [3] and more recently inverted internal limiting membrane flap application [4] or autologous neurosensory retinal free flap transplantation [5].

Here we present a refractory large macular hole after repeated attempts with ILM peeling and secondary silicone oil installation, which remained open. In this case we used a novel technique by the application of subretinal fluid to shift the released retina towards the center to achieve an anatomical closure of the macular hole.

## Case presentation

**Fig. 1:**
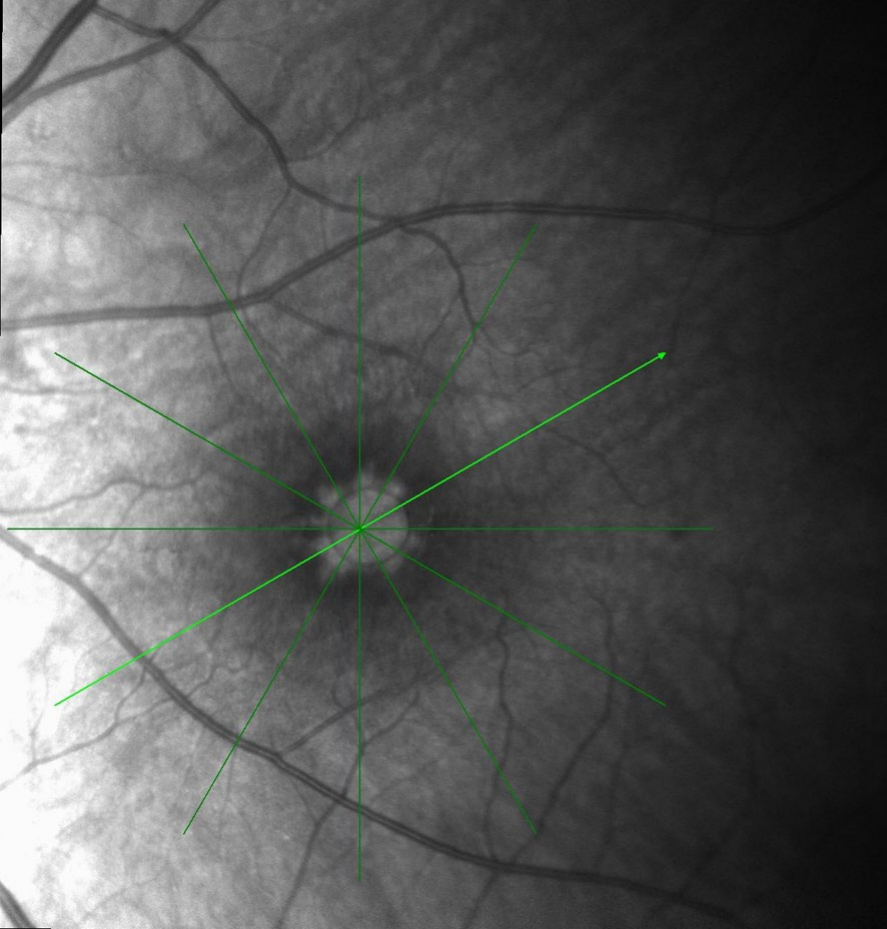
Fundus photo of the giant macular hole with radial scans, measureing a diameter of 1444–1376 μm

**Fig. 2:**
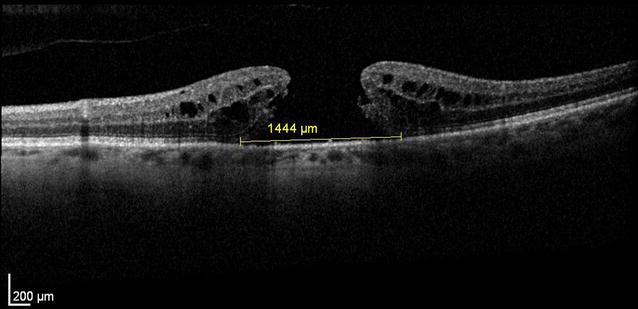
Corresponding SD-OCT: Prior to surgery there was a refractory giant FTMH significant intraretinal edema and a horizontal diameter of 1444 μm

A 83 years old patient presented a large sized FTMH in his left eye. Uneventful ppV with consecutive chromovitrectomy, application of brilliant blue dye (BBD) and consecutive 12% C2F6 gas application remained unsuccessful. Although the postoperative optical coherence tomography (OCT) defined no residual epiretinal membrane on the retina, the macular hole remained unchanged (horizontal 1444 μm vertical 1380 μm microns diameter). During reoperation we confirmed this observation by restaining the retinal surface by BBD and determined a negative staining up to the upper and lower arcade. Due to the extensive ILM removal during the first surgery, additional ILM patch transplantation was not feasible in this situation anymore and a silicone oil tamponade was installed for 6 week. However, the macular hole remained also unchanged on postoperative OCT and after oil removal (Figs. 1, 2). In a résumé, we discussed with the patient the past procedures and additional published treatment options. In preparation for an additional reoperation we remembered, that our previous application of subretinal fluid during limited macular translocation was capable to shift the entire retina up to 1700 μm [6]. Based on this experience with macular translocation we adopted this concept for our patient and developed a novel surgical approach for macular hole surgery. The patient agreed and gave informed consent for this procedure.

First, we installed a small decaline bleb over the macular hole to cover the edges of the hole. Next, we carefully applied three small subretinal blebs of 2–3 DD in the superior, temporal and inferior quadrant using a 41-gauge subretinal cannula connected to 1 cc syringe filled with balanced salt solution (BSS). The next step was the removal of the decaline by shifting the bleb over the optic disc and removing it safely. Then we applied BSS fluid at one retinotomy until the subretinal blebs connected to a small perifoveal serous neurosensory detachment. The released paracentral retina was gently massaged towards the foveal center using the Tano diamond dusted scraper.

After fluid air exchange, we installed silicon oil to secure the healing of the sealed macular hole, and to observe the retina during the early postoperative period. Five days after surgery we observed by OCT a closure of the giant macular hole.

**Fig. 3:**
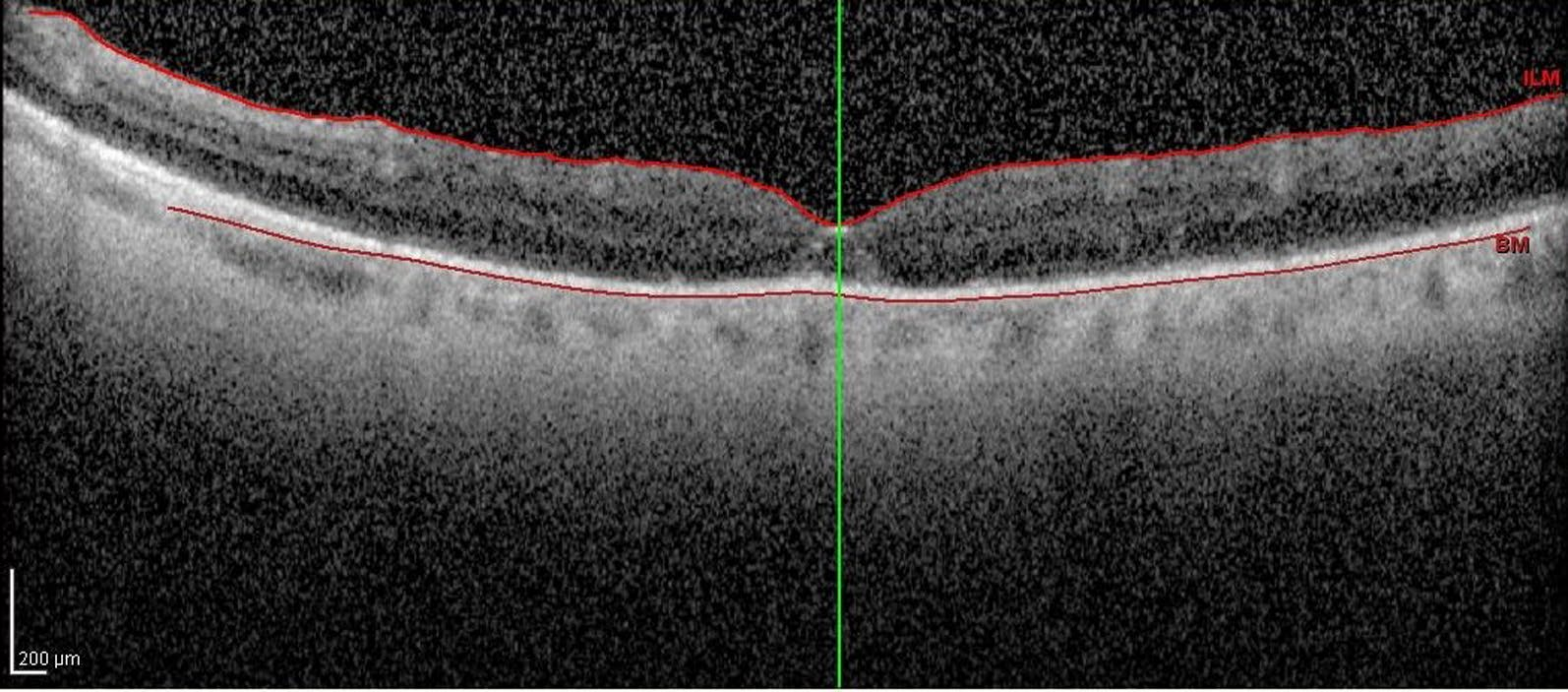
SD-OCT demonstrated 5 days after surgery the closure of the FTMH and restoration of the anatomical architecture

Moreover, there was a restoration of the anatomical architecture (Fig. 3) with significant visual acuity improvement to 20/200 postoperatively.

## Discussion

Numerous secondary surgical attempts have been described to achieve a surgical closure of refractory FTMH after successful chromovitrectomy with ILM removal: (a) while restaining the retinal surface may confirm the complete release of the ILM, (b) repeated gas or silicone oil application may enhance the duration of the tamponade and time to close especially large holes. (c) More recent ILM patches may release growth factors or guide as sheath to the adjacent retina to heal the macular hole [4]. (d) The contraction of the macular hole itself may be enhanced by the cellular force of platelet cell application, or intraoperative aspiration at the edges of the macular hole with some fluid or by a forceps [7]. However, the mechanical or tactile traction may also traumatize the delicate retinal structure limiting the functional outcome and visual acuity. (e) Here we present a novel approach, which involves the induction of a serous macular detachment around the MH and parafoveal retinal massage to narrow the edges and to achieve an anatomical closure.

We hypothesize, that in giant FTMH not necessarily the release of the tangential traction, but the firm adhesion of the perpendicular photoreceptors to the RPE may prevent a successful closure of the macular hole. In limited macular translocation we learned that the retina may shift by more than 1700 μm (range, 680–3200) by creating a serous detachment and scleral imbrication.

Massaging the neurosensory retina with a scrapper is also potentially a traumatic maneuver. Controversly, we discussed if the mechanical brushing of the parafoveal retinal surface with a Tano scraper is essential and required in all cases. An iatrogenic trauma may be induced by the extend of the epiretinal massage. Thus, we proposed in future cases with primary failed larger FTMH closure, to apply solely subretinal fluid with a gas endotamponade in order to close even larger macular holes without secondary mechanical maneuvers.

## Conclusion

A primary release of the centripetal force by the ILM removal, followed by a release of the RPE-photoreceptor adherence may mobilize the elastic retina from epiretinal and subretinal adhesions completely. Secondary, stretching of the retina by application of subretinal fluid and tactile massage may enlarge the retinal surface covering potentially large macular holes. The here presented novel subretinal approach may serve as a first prove of principle to close residual FTMHs after subretinal fluid injection successfully.
